# Supplementary material for: Fabrication and Characterization of Magnetic Cellulose–Chitosan–Alginate Composite Hydrogel Bead Bio-Sorbent
Source: Polymers (Basel). 2023 May 29;15(11):2494. doi: 10.3390/polym15112494 (PMC10255174; doi:10.3390/polym15112494)
Supplement: Supplementary file 1 [file polymers-15-02494-s001.zip › polymers-2385352-supplementary.pdf]

Supplementary Materials

Fabrication and Characterization of Magnetic Cellulose–Chitosan–Alginate Composite Hydrogel Bead Bio-Sorbent

Aida Syafiqah Abdul Rahman [1], Ahmad Noor Syimir Fizal [2], Nor Afifah Khalil [1], Ahmad Naim Ahmad Yahaya [3], Md. Sohrab Hossain [4] and Muzafar Zulkifli [3,*]

1 Malaysian Institute of Chemical and Bioengineering Technology, Universiti Kuala Lumpur Branch Campus, Alor Gajah 78000, Melaka, Malaysia; aida.syafiqah@s.unikl.edu.my (A.S.A.R.); nafifah.khalil@s.unikl.edu.my (N.A.K.)

2 Lebuh Persiaran Tun Khalil Yaakob, Centre for Sustainability of Ecosystem & Earth Resources (Pusat ALAM) Universiti Malaysia Pahang, Gambang 26300, Pahang, Malaysia; syimir@umpholdings.my

3 Green Chemistry and Sustainability Cluster, Branch Campus, Malaysian Institute of Chemical and Bioengineering Technology Universiti Kuala Lumpur, Taboh Naning, Alor Gajah 78000, Melaka, Malaysia; ahmadnaim@unikl.edu.my

4 HICoE-Centre for Biofuel and Biochemical Research, Institute of Self-Sustainability Building, Fundamental and Applied Sciences Department, Universiti Teknologi PETRONAS (UTP), Seri Iskandar 32610, Perak, Malaysia; sohrab.hossain@utp.edu.my

* Correspondence: muzafar@unikl.edu.my

S1: Moisture content and mass content of materials present in CeMA, CMA, and CCM

**Hydrogel Bead**	**Mass ratio of cellulose: chitosan: magnetite: alginate**	Cellulose content (mg)	Chitosan content (mg)	Magnetite content (mg)	Alginate content (mg)	Total mass content (mg)	Weight of wet individual hydrogel bead (mg)	Weight of dry individual hydrogel bead (mg)	Moisture content dry basis (%)
**CeMA**	**1: 0: 0.1: 2**	0.67	NA	0.067	1.33	2.07	33.00	2.80	1101.21
**CMA**	**0: 1: 0.1: 2**	NA	0.67	0.067	1.33	2.07	31.80	2.80	1023.53
**CCMA**	**1: 1: 0.1: 2**	0.67	0.67	0.13	1.33	2.80	41.40	4.00	935.83

NA: Not Available

S2: Calculations for the mass content of materials in CeMA, CMA, and CCMA

Total of hydrogel beads produced: 3,000 hydrogel beads / 200 mL of solution

Material	Cellulose	Chitosan	Magnetite	Alginate
Total mass of material (g)	2 g	2 g	0.2 g	4 g

The mass content of materials per one hydrogel bead is calculated by using equation below:

$$\text{Mass content of material per hydrogel bead (g)} \equiv \frac{\text{Total mass of material}}{\text{3,000 Beads}}$$

S3: The IR spectra of possible functional groups in cellulose, chitosan, magnetite, and alginate based on previous studies ([11,12,21,32])

Sample	Functional group	Stretching/ Vibration	Peak appearance	Wavelength (cm^{-1})	**CCMA**
Alginate	Carboxylate anion, C=O and C-O	Strong asymmetrical and weak symmetrical	Strong and weak peak	1650 - 1550 and near 1400	**1595**<br>**1417**
Chitosan and Cellulose	Amines and Hydroxyl, N-H and O-H	Overlapping of N-H and O-H	Broad and strong peak	3330 - 3060	**3294**
Chitosan	Amide, NH_2	Asymmetrical and symmetrical of N-H stretching	Strong peak	1650 - 1515	**1595**
Magnetite	Fe-O	Fe-O bond	Strong peak	533	**-**
Cellulose Chitosan Alginate	Ether, C-O-C	Symmetrical C-O-C stretch	Strong peak	1075 - 1020	**1025**
